# Bigel Formulations of Nanoencapsulated St. John’s Wort Extract—An Approach for Enhanced Wound Healing

**DOI:** 10.3390/gels9050360

**Published:** 2023-04-25

**Authors:** Yoana Sotirova, Viliana Gugleva, Stanila Stoeva, Iliyan Kolev, Rositsa Nikolova, Maria Marudova, Krastena Nikolova, Yoana Kiselova-Kaneva, Minka Hristova, Velichka Andonova

**Affiliations:** 1 Department of Pharmaceutical Technologies, Faculty of Pharmacy, Medical University of Varna, 9000 Varna, Bulgaria; 2 Department of Pharmacology, Toxicology and Pharmacotherapy, Faculty of Pharmacy, Medical University of Varna, 9000 Varna, Bulgaria; 3 Department of Pharmaceutical Chemistry, Faculty of Pharmacy, Medical University of Varna, 9000 Varna, Bulgaria; 4 Institute of Mineralogy and Crystallography, Bulgarian Academy of Sciences, Acad. G. Bonchev, 1113 Sofia, Bulgaria; 5 Department of Physics, Faculty of Physics and Technology, University of Plovdiv “Paisii Hilendarski”, 4000 Plovdiv, Bulgaria; 6 Department of Physics and Biophysics, Faculty of Pharmacy, Medical University of Varna, 9000 Varna, Bulgaria; 7 Department of Biochemistry, Molecular Medicine and Nutrigenomics, Faculty of Pharmacy, Medical University of Varna, 9000 Varna, Bulgaria; 8 Department of Physiology and Pathophysiology, Faculty of Medicine, Medical University of Varna, 9000 Varna, Bulgaria

**Keywords:** biphasic gels, hyperforin, *Hypericum perforatum*, nanostructured lipid carriers, wounds

## Abstract

This study aimed to develop a semisolid vehicle for topical delivery of nanoencapsulated St. John’s wort (SJW) extract, rich in hyperforin (HP), and explore its wound-healing potential. Four nanostructured lipid carriers (NLCs) were obtained: blank and HP-rich SJW extract-loaded (HP-NLC). They comprised glyceryl behenate (GB) as a solid lipid, almond oil (AO), or borage oil (BO) representing the liquid lipid, along with polyoxyethylene (20) sorbitan monooleate (PSMO) and sorbitan monooleate (SMO) as surfactants. The dispersions demonstrated anisometric nanoscale particles with acceptable size distribution and disrupted crystalline structure, providing entrapment capacity higher than 70%. The carrier exhibiting preferable characteristics (HP-NLC2) was gelled with Poloxamer 407 (PM407) to serve as the hydrophilic phase of a bigel, to which the combination of BO and sorbitan monostearate (SMS) organogel was added. The eight prepared bigels with different proportions (blank and nanodispersion-loaded) were characterized rheologically and texturally to investigate the impact of the hydrogel-to-oleogel ratio. The therapeutic potential of the superior formulation (HP-NLC-BG2) was evaluated in vivo on Wistar male rats through the tensile strength test on a primary-closed incised wound. Compared with a commercial herbal semisolid and a control group, the highest tear resistance (7.764 ± 0.13 N) was achieved by HP-NLC-BG2, proving its outstanding wound-healing effect.

## 1. Introduction

Intact skin integrity is an important factor, contributing to the barrier and protective properties of the human epidermis. St. John’s wort (*Hypericum perforatum* L., Clusiaceae (Hypericaceae); SJW) has been topically applied in the therapy of different skin conditions (e.g., (sun)burns, wounds, and superficial injuries), owing to one of its major constituents—the polyprenylated phloroglucinol hyperforin (HP) [1]. In contrast to other phytochemicals with antimicrobial, anti-inflammatory, and antioxidant properties [2,3], HP’s intrinsic photo and oxygen sensitivity [4] limit its wider topical use. The latter motivates its inclusion in a nanoscale system to serve as a protective reservoir and preserve its chemical stability. With regard to the lipophilic nature of HP [5], nanostructured lipid carriers (NLCs) can be considered suitable for this purpose.

NLCs have been widely used as dermal drug delivery systems due to their beneficial characteristics: biocompatibility, biodegradability, controlled release profile of encapsulated active pharmaceutical ingredients (APIs), and occlusive properties, promoting skin hydration and, thus, drug permeation and bioavailability [6,7,8,9]. NLCs are obtained by blending solid and liquid lipids, which account for their disordered, “imperfect” matrix. This structure determines their higher drug-loading ability (compared to solid lipid nanoparticles) and improved drug retention [10]. Various phenolic compounds have been encapsulated in NLCs, thereby obtaining enhanced solubility of the APIs (ellagic acid NLCs), reduced skin irritation and photoprotection (protocatechuic acid NLCs), and improved skin penetration and bioavailability (resveratrol NLCs) [11,12,13]. Nevertheless, the topical effects of NLCs may be further improved when sufficient skin contact time is provided, e.g., through their inclusion in a semisolid vehicle. 

Gels are among the most often exploited semisolid dosage forms for dermal application. They structurally represent a three-dimensional network with liquid molecules entrapped between its units [14]. On the basis of the nature of the employed solvent, they are classified into two major groups: hydrogels, containing polar liquids (i.e., purified water [15]), and organogels, comprising nonpolar solvents, typically mineral or vegetable oils [16]. The good compatibility and spreadability of hydrogels, as well as their hydrating and skin-cooling properties, define their broad application and patient acceptance [17]. Some of their disadvantages, e.g., low solubilizing/delivery capacity for hydrophobic drugs and limited skin permeation, can be successfully overcome by organogels, but with the major drawback of a greasier consistency [18]. In an attempt to elaborate a vehicle with optimal characteristics, novel semisolid formulations named bigels have been developed. They are stable hybrid structures obtained by mixing hydrogels and organogels in different proportions [18,19]. These biphasic formulations can overcome their components’ main limitations by combining their advantages; their easy application and removal results in improved compliance, and their ability to carry different-natured APIs simultaneously ameliorates drug penetration through the stratum corneum [18,20]. 

The herein-described NLC dispersions comprise glyceryl behenate (GB) as a solid lipid, almond oil (AO) or borage oil (BO) as the liquid lipid, and a surfactant blend of polyoxyethylene (20) sorbitan monooleate (PSMO) and sorbitan monooleate (SMO). GB, a mono-, di-, and tribehenin mixture, is a chemically stable amphiphilic glyceride with a partially crystalline structure. These characteristics determine its ability to provide sufficient space for drug encapsulation [21,22]. The selection of AO or BO as the liquid lipid component of NLCs is based on several factors: their natural origin, compatibility with the skin’s lipophilic constituents, capacity to improve drug penetration, and ability to provide a moisturizing effect [23]. In order to achieve increased physical stability of nanoparticulate dispersions, a mixture of the biocompatible PSMO and SMO was used [24,25]. 

The bigels developed in the current study were composed of sorbitan monostearate (SMS) as an organogelator and BO (nonpolar medium), representing the oleogel fraction, and an aqueous phase (purified water/ HP-NLC), gelled with Poloxamer 407 (PM407). SMS is a non-ionic surfactant obtained by esterification of stearic acid and sorbitol. Its hydrophilic–lipophilic balance (HLB) value of 4.7 determines its primary application in pharmaceutics as a water-in-oil emulsifier [26]. In bigels, it is used due to its capability to immobilize nonpolar liquids, forming fluid matrix oleogel systems [18]. BO was selected as an oil phase for the organogel on the grounds of its numerous skin-beneficial effects. Produced from the seeds of the *Borago officinalis* plant, BO is considered one of the main sources of γ-linolenic acid (GLA), constituting between 20% and 25% of the oil [27]. In addition to GLA, the oil contains saturated and unsaturated fatty acids, the most abundant of which are linoleic (~33%), oleic (~20%), palmitic (10–12%), and stearic (~4%) acids [28]. BO has been used in the therapy of various degenerative and cardiovascular diseases and dermal conditions such as seborrheic and atopic dermatitis [29,30]. In bigel formulations, its favorable skin-related properties would complement the beneficial effects of NLCs. The hydrogel fraction of the elaborated semisolids contains a mixture of nanocarriers and purified water, representing the aqueous medium, and PM407 as a gelling agent. The latter is a synthetic polymer comprising polyethylene oxide and polypropylene oxide chains, primarily used as a solubilizer and emulsifier because of its surface-active properties [31]. After a certain concentration, PM407 monomers are able to self-aggregate into micellar structures, which, by interacting with each other, form a hydrogel [32].

The current study aims to establish a suitable combination of a colloidal carrier (NLC) and a semisolid platform (bigel), allowing topical application of an HP-rich SJW extract. First, after physicochemical, structural, and morphological characterization, the NLC model, which possessed favorable properties, was selected to serve as a protective reservoir of HP-rich SJW extract (HP-NLC). Next, bigels with different hydrogel-to-organogel ratios were developed, and their textural and rheological properties were evaluated. After determining the superior biphasic formulation among all investigated, in vivo studies were conducted to assess its wound-healing potential.

To the best of our knowledge, there are no data on the incorporation of St. John’s wort extract, rich in hyperforin, in nanostructured lipid carriers. Accordingly, the inclusion of such colloidal systems in biphasic gels for dermal application and wound treatment has not been explored either.

## 2. Results and Discussion

### 2.1. HPLC-UV Analysis and HP Quantification

For the HP standard, linearity in the range of 1.0–50.0 μg/mL (after sixfold analysis) was established from the straight-line equation (y = 0.3028x – 0.0053) and the correlation coefficient (R^2^ = 0.9999). The LOQ of the analyte was found to be 1.0 μg/mL [33]. Next, the HPLC method was used to assess the implemented extraction technique, considering the relative HP concentration.

The maceration method was expediently applied to prepare the SJW extract as an especially suitable technique for isolating thermolabile natural substances, such as HP [34,35]. In order to reduce the consumption of organic solvent and the time for its subsequent distillation, very close solvent-to-solid ratios were chosen in absolute value, from 2.5:1 to 3.33:1 (*v*/*w*). The removal of the present oxygen from the macerate medium, on the other hand, necessitated the performance of an additional argon purging step—another measure that should prevent the destruction of in situ “released” HP [36].

**Figure 1:**
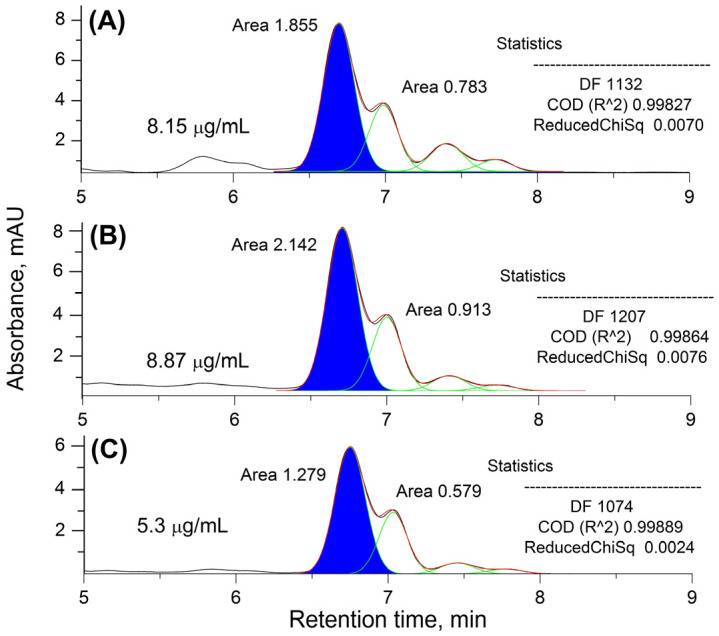
Comparative HPLC analysis of a commercial St. John’s wort (SJW) CO_2_ extract, rich in hyperforin (HP) (**A**), a maceration-obtained extract at a solvent-to-solid ratio of 3.33:1 (**B**), and a maceration-obtained extract at a solvent-to-solid ratio of 2.5:1 (**C**). The deconvoluted chromatographic peaks are presented in green, and the significant (HP) peaks are filled with blue; their superposition patterns are represented in red. The degrees of coincidence of the latter with the real chromatograms were statistically assessed; the results obtained are presented in each subfigure.

The extraction yield of the newly introduced extraction protocol was chromatographically evaluated. The performed HPLC analysis revealed that, in both cases, a high extraction yield was achieved (>5.0 μg/mL; Figure 1B,C). Logically, as the amount of solvent used increased, the degree of extraction of the principal acylphlorogluside also increased (from 5.3 to 8.87 μg/mL). Regarding the content of the main prenylated phloroglucinol, the quality of the herein obtained extract was found to be comparable to that of the commercially available product (Figure 1A,B).

### 2.2. Characterization of the Nanocarriers 

#### 2.2.1. Particle Size, Polydispersity Index, Zeta Potential, and Entrapment Efficiency

The developed blank nanodispersions were whitish, milky, homogeneous liquids, while HP-NLC systems possessed the extract’s greenish shade. The NLC systems demonstrated particle sizes (Z-average) in the nanoscale (Table 1), with polidisperisty index (PI) indicating an acceptable homogeneity [37]. Combined with the favorable zeta potential (ZP) values (higher than |30| mV), good physical stability of the nanocarriers could be expected [38]. The addition of the HP-rich SJW extract led to the formation of particles with larger dimensions while simultaneously not altering their surface electrical charge. Furthermore, a successful incorporation of the extract may be considered valid for both carriers as they presented entrapment efficiency (EE) values higher than 60% [39]. However, the BO-based system displayed a significantly higher encapsulation ability.

After 1 month of storage at 4 °C, no visual changes in the dispersions, e.g., coalescence, phase separation, or gelling, were reported. The mean particle size of all samples was increased, but a slightly deteriorated uniformity (demonstrated by the higher PI) was observed only in HP-NLC1. Intriguingly, the ZP of the nanoreservoirs remained unaffected, excluding HP-NLC2, where the values of this parameter were elevated. More importantly, the BO-containing system exhibited higher EE after the research period.

#### 2.2.2. Attenuated Total Reflectance Fourier-Transform Infrared Spectroscopy

**Figure 2:**
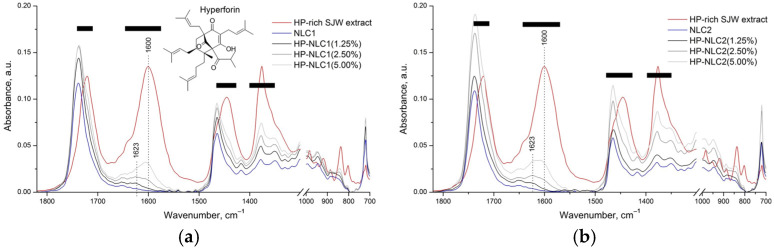
Attenuated total reflectance Fourier-transform infrared (ATR-FTIR) of the isolated HP-rich SJW extract (red line), blank colloidal dispersions (NLC1 and 2; blue lines), and their composites: samples with common codes HP-NLC1 and 2 (represented by grayscale lines). For the readers’ convenience, the spectra of the forms based on almond oil (AO) and borage oil (BO) are presented in separate subfigures: (**a**) and (**b**), respectively.

All IR absorption spectra are presented in a relatively narrow (from 1850 to 700 cm^−1^) but quite informative spectral subregion of the mid-infrared spectrum (Figure 2). Furthermore, additional HP-NLC samples with higher extract contents (2.50% and 5.00%) were obtained to verify some basic qualitative analytical results.

The first, most direct evidence for the successful inclusion of the HP-rich SJW extract was the general appearance of HP-NLC1 and 2 (1.25%) IR spectra (Figure 2). Indeed, in certain spectral regions (marked with black bars), the spectra of the samples in question acquired the “genetic” marks of their constituents—NLC dispersions and the extract. The most informative of all, however, is the area of C=C vibrations from 1600 to 1630 cm^−1^, wherein IR radiation was absorbed only by HP-rich SJW extract. This is why the incorporation of the latter in the composition of NLC dispersions at 1.25% (*w*/*w*) caused the appearance of barely noticeable absorption bands with maxima at 1623 cm^−1^. The same regularity was observed in the spectra of the remaining samples (HP-NLC1 (2.50%), HP-NLC1 (5.00%), HP-NLC2 (2.50%), and HP-NLC2 (5.00%)), but with the expected quantitative changes in the intensity of the band in question (Figure 2). Along with this, however, the appearance of an additional band with a maximum at 1600 cm^−1^ (characteristic of the plant extract itself) was also registered in the spectra of the extract-overloaded samples.

The reported spectral dependencies can be used as real proof of the successful incorporation of the HP-rich SJW extract in NLC particles (at 1.25%), which, if introduced in higher amounts, forms co-aggregates (real mixtures) with HP-NLC dispersions.

#### 2.2.3. X-ray Diffraction Analysis

**Figure 3:**
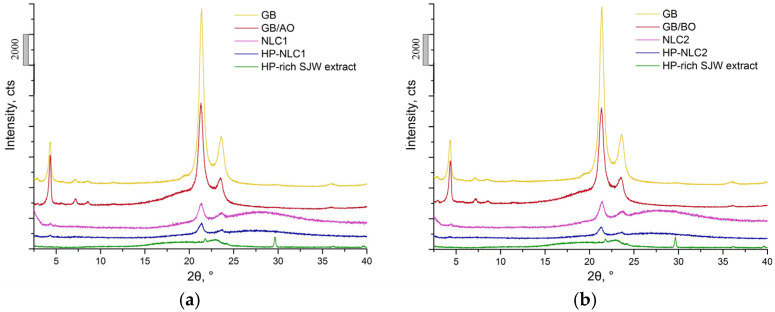
X-ray diffraction (XRD) patterns of the used solid lipid (glyceryl behenate; GB), the resulting lipid blends (GB with almond oil (GB/AO) and GB with borage oil (GB/BO)), and nanoparticulate dispersions (NLCs and HP-NLCs). The green line illustrates the X-ray diffractogram of HP-rich SJW extract. The individual samples are presented in an identical manner to that in Figure 2, namely: in subfigure (**a**)—the diffractograms of the AO-based forms, and in subfigure (**b**)—those of the BO-based ones.

The performed X-ray diffraction (XRD) measurements proved the partially crystalline structure of the solid lipid used [21]. The observed reflexes at 21.37° and 23.59°, as well as the corresponding d-spacing values (0.42 and 0.38 nm, respectively), revealed the major presence of the β’ polymorphic form in all GB-based samples (Figure 3) [40]. 

The introduction of the liquid lipid was expected to cause a reduction in the degree of GB crystallinity [41]. Indeed, the intensity of the reflections in question was significantly decreased in the diffractograms of the two lipid mixtures (GB/AO and GB/BO). In this regard, NLCs and HP-NLCs could be characterized as structures with an even more disrupted lattice order [42]. Furthermore, the diffraction peaks characteristic of the HP-rich SJW extract (at 21.80° and 29.65°) were not present in the HP-NLC systems; hence, the latter was presumably in an amorphous state or molecularly dispersed within the lipid matrix of the nanocarriers [43,44,45].

#### 2.2.4. Transmission Electron Microscopy Investigations

**Figure 4:**
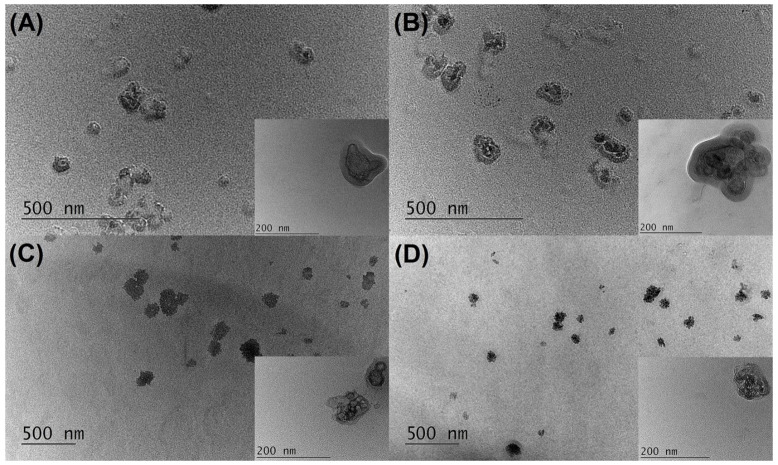
Transmission electron microscopy (TEM) images of NLC1 (**A**), HP-NLC1 (**B**), NLC2 (**C**), and HP-NLC2 (**D**).

Transmission Electron Microscopy (TEM) observations revealed that NLC and HP-NLC samples mainly comprised irregularly shaped, well-delimited particles (Figure 4). Furthermore, the analysis confirmed their nano-ranged mean size, as reported by the dynamic light scattering (DLS) measurements. The presence of particle aggregates in NLC1 and HP-NLC1 was more pronounced despite their lower polydispersity indices. Overall, the clear core–shell structure of the blank nanoparticles was less distinct after the inclusion of the HP-rich SJW extract. However, the encapsulation did not alter the integrity of the nanocarriers.

On the grounds of the greater entrapment capability combined with desirable PI and ZP values, the HP-NLC2 formulation was chosen as a carrier of HP-rich SJW extract for subsequent incorporation into a semisolid vehicle. 

### 2.3. Characterization of the Bigels

#### 2.3.1. Physical Appearance

**Figure 5:**
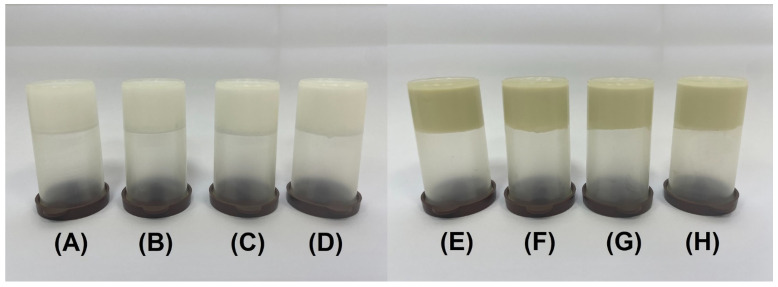
Tube inversion test of blank (BG1 (**A**), BG2 (**B**), BG3 (**C**), and BG4 (**D**)) and HP-NLC-loaded (HP-NLC-BG1 (**E**), HP-NLC-BG2 (**F**), HP-NLC-BG3 (**G**), and HP-NLC-BG4 (**H**)) bigels.

Once the preferred nanodispersion was selected, the research continued with the preparation of biphasic gels. The elaborated semisolids were whitish (blank) or greenish (HP-NLC-loaded), all having a slight scent of borage oil and a creamy texture (Figure 5). The tube inversion test confirmed gel formation for all eight formulations. BG4 and HP-NLC-BG4 were characterized by greasy consistency, and, to avoid developing oily-natured bigels, they were excluded from the study.

#### 2.3.2. Optical Microscopy

**Figure 6:**
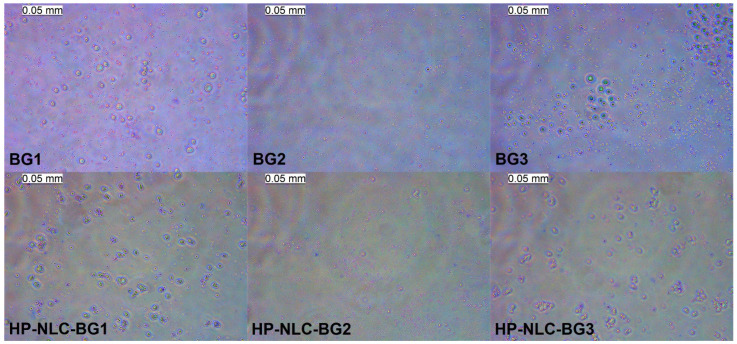
Optical microscopy of blank and HP-NLC-loaded semisolids.

The micrographs revealed formulations with differently shaped organogel droplets dispersed into the hydrogel medium (Figure 6). A nonuniform distribution, including droplets with dimensions larger than 5 μm, was presented by BG1 and 3; in contrast, BG2 was visibly more homogeneous. Regarding HP-NLC-containing semisolids, better uniformity was again observed in the formulation comprising 20% oleogel, whereas HP-NLC-BG1 and 3 possessed droplet aggregates.

#### 2.3.3. pH Analysis

The pH analysis was conducted to assess the skin tolerance of the elaborated bigels. Regarding the slightly acidic medium of the skin surface, applying formulations possessing similar pH values is recommended [46]. All investigated semisolids showed physiologically tolerable pH values (Table 2). The addition of nanoparticulate dispersion caused an increase in acidity; however, the pH values of the HP-NLC-loaded bigels did not suggest skin irritation.

#### 2.3.4. Centrifugation Test

**Figure 7:**
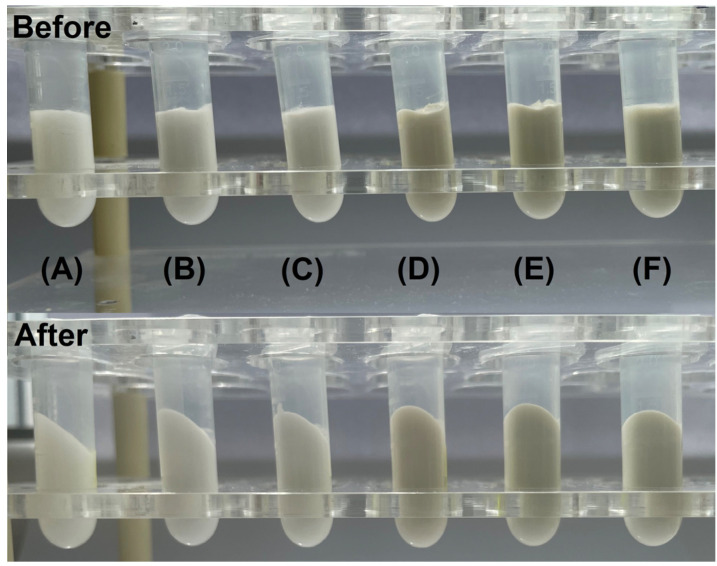
Visual appearance of blank (BG1 (**A**), BG2 (**B**), and BG3 (**C**)) and HP-NLC-loaded (HP-NLC1 (**D**), HP-NLC-BG2 (**E**), and HP-NLC-BG3 (**F**)) bigels before and after centrifugation test.

A centrifugation test was conducted to explore the possibility of phase separation occurring with the application of mechanical force [47]. No visual changes were observed after two cycles were performed (Figure 7). Therefore, blank and HP-NLC-loaded bigels could be characterized as having strength, enough to remain unaffected after this accelerated stability study.

#### 2.3.5. Spreadability

Assessing the spreadability of the topical semisolids is crucial; this is related to the uniformity of distribution on the skin, which can affect the efficiency of the therapy [48]. The developed formulations exhibited spreading diameters in the range of 24.75–31.5 mm (Table 3); according to Lardy et al. [49], they could be described as very stiff (Ø < 40 mm). 

The spreading diameters of all HP-NLC-loaded bigels were smaller than those of the corresponding blank formulations. The latter is probably based on the hydrogel base’s higher viscosity, owing to the lipid nanodispersion’s presence. The statistical difference between the spreading diameters of BG1 and 3, as well as between those of HP-NLC-BG1 and 3, suggested that an increase in the oleogel fraction by 10% did not significantly affect this parameter. Therefore, the formulations containing hydro- and oleogel in proportions of 80/20 represented a limit point above which the spreadability of the semisolids was diminished.

#### 2.3.6. Textural Analysis

**Figure 8:**
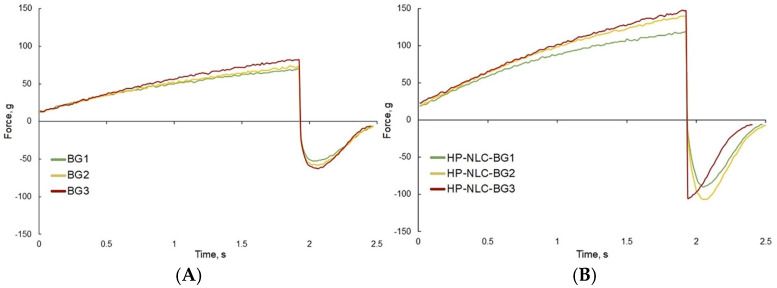
Force–time curves of blank (**A**) and HP-NLC-loaded bigels (**B**) obtained from the textural analysis.

Firmness is a characteristic used to describe the resistance of a given substance to localized deformation. It can be measured by the peak force determined during a single compression test [50,51]. All three HP-NLC-loaded bigels showed higher firmness values than the corresponding blank formulations (Table 3, Figure 8). As with the spreadability, the latter could be attributed to the higher viscosity of the nanodispersion-containing hydrogel base. Considering blank semisolids, the increasing oleogel quantity caused a significant change in the firmness only when it reached 30%. In contrast, HP-NLC-loaded bigels demonstrated rising firmness values with each 10% oleogel increase.

Cohesiveness is a measure of the work required to produce a deformation during single compression. Graphically, it is represented by the positive area under the force–time curve. Since firmness and cohesiveness are closely related [52], similar conclusions could probably be expected here. Again, HP-NLC-loaded formulations showed higher values than the corresponding blank semisolids. However, the development of more cohesive structures with the increasing oleogel content was observed only in nanosuspension-containing bigels. 

The negative area under the force–time plot illustrates the adhesiveness, i.e., the force necessary to “break” the attractive interaction between the formulation and the probe [52]. This parameter corresponds to the ease of removing an adhered substance from a given material; hence, it can be interpreted as the adhesion rate on the skin in reference to topical semisolids [53]. The presence of the HP-NLC dispersion caused a rise in the absolute value of semisolid adhesiveness. The results of the blank formulations were comparable, but the difference between HP-NLC-BG1 and 3 reaffirmed the significance of the 80:20 hydrogel-to-oleogel ratio. The 20% lipophilic fraction content resulted in more adhesive bigels, while its further increase did not cause any alteration.

#### 2.3.7. Rheological Studies

Among all rheological models studied, the most applicable for BG1 and 3 was the Herschel–Bulkley model (presented with a coefficient of determination of R^2^ = 0.99; Table 4). 

**Figure 9:**
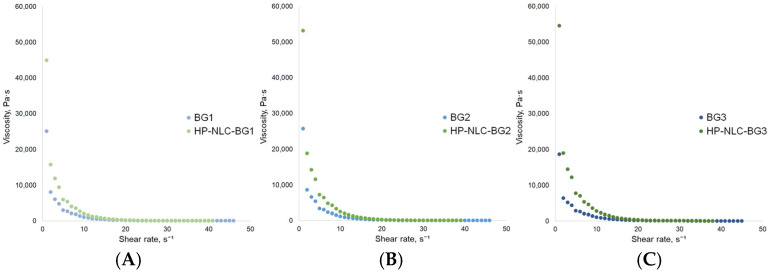
Effect of shear rate on the viscosity of BG1 and HP-NLC-BG1 (**A**), BG2 and HP-NLC-BG2 (**B**), and BG3 and HP-NLC-BG3 (**C**).

The measured yield stress values (283.59 ± 4.41 Pa for BG1 and 236.02 ± 8.64 Pa for BG3) suggested that less work was needed to make the oleogel-richer bigels flow. The formulations’ low power law indices (*n* < 1) demonstrated their shear-thinning behavior. The latter was confirmed by the graphically presented decrease in the dynamic viscosity with the increase in shear rate (Figure 9A,C). Moreover, considering that better gel consistency relates to higher K values and a lower flow index [54], BG3 possessed a preferable texture.

The obtained zero yield stress value for BG2 indicated that this formulation flowed instantly after a stress application. In this regard, the Ostwald de Waele model characterized its behavior better, despite the relatively low coefficient of determination (R^2^ = 0.72). The power law index of BG2 was also below unity; hence, the studied bigel demonstrated pseudoplastic flow (illustrated in Figure 9B).

Similarly, the Herschel–Bulkley model presented the highest coefficient of determination for HP-NLC-loaded bigels (Table 5). These formulations can also be characterized as shear-thinning fluid according to their low *n* values and the relationship between the dynamic viscosity and the shear rate (Figure 9). Here, the rising yield stress values suggested that an increase in the oil phase led to the formation of more rigid structures. HP-NLC-BG2 showed the lowest flow index and the highest K value. These results reinforced its significance as a threshold point at which superior consistency was also achieved.

On the basis of all results obtained, HP-NLC-BG2 was preferred as a semisolid vehicle of the nanoencapsulated SJW extract.

### 2.4. In Vivo Wound-Healing Effect

In this study, the therapeutic potential of the selected bigel was evaluated and compared to that of a commercial semisolid formulation and to a negative control. The selection of the reference product was based on its herbal content and the expected therapeutic outcome: the plants contained therein have been traditionally used for their wound-healing effect [55,56,57,58].

The healing of the primary-closed incisions was estimated through the tensile strength test. Among the variety of biomechanical methods determining the extent of skin wound recovery, the assay mentioned above stood out as one of the easiest for implementation [59]. 

**Figure 10:**
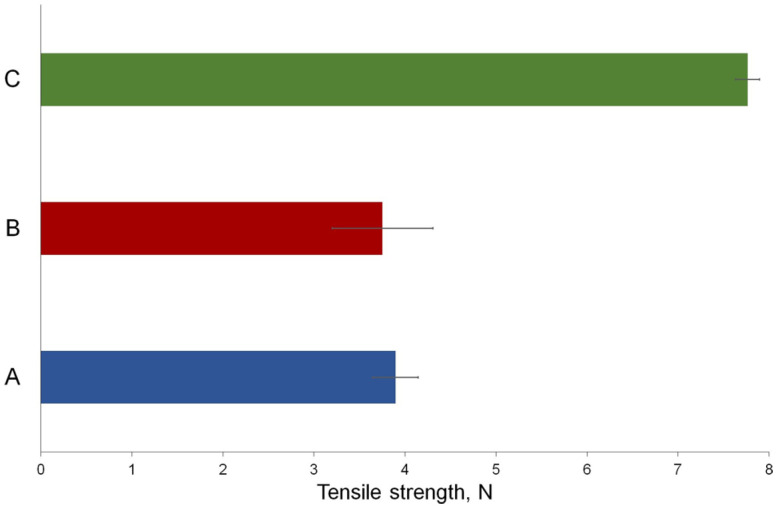
Tensile strength results for the control group (**A**), the reference group (**B**), and the experimental group (**C**).

The average values of strength needed to interrupt regenerated skin’s integrity (given in N with calculated standard deviation (SD)) were as follows: 3.895 ± 0.25 for the negative control group, 3.753 ± 0.55 for the reference group, and 7.764 ± 0.13 for the HP-NLC-BG2-treated group. Prima facie, the force necessary for breaking the healed wounds of animals treated with the biphasic formulation was almost twice as much as that needed for the wounds of animals in the remaining two groups (Figure 10). 

Table 6 presents a statistically significant difference between the values obtained in the control and experimental groups and between the latter and the reference groups. These results demonstrated that enhanced skin repair, estimated through the rupture resistance of the recovered wounds, was achieved only after HP-NLC-BG2 application.

## 3. Conclusions

In this study, two established NLC models, differing in the liquid lipid they contained (i.e., almond or borage oil), were loaded with a newly obtained SJW extract rich in HP. After incorporation of the latter, the carriers maintained their dimensions in the nano-range below 150 nm, with high surface charge values (>|30| mV) and acceptable uniformity (PI < 0.3). The encapsulation of the extract, considered prosperous through the high EE values evaluated (>70%), was additionally proven by ATR-FTIR analysis. Moreover, HP-NLC systems presented particles with a more disordered inner structure, probably related to the reduced degree of crystallinity of the solid lipid used. The model showing preferable characteristics after 1 month of storage at 4 °C (HP-NLC-2) was chosen as a delivery platform for HP-rich SJW extract. In order to ensure sufficient skin contact time, the nanodispersion was incorporated into a semisolid vehicle prior to topical application.

As an attractive research field in terms of drug delivery, bigels were exploited as a semisolid carrier of the selected nanoparticulate dispersion. Six differently proportioned bigels (blank and HP-NLC-loaded), nonoily and tailored to the skin’s pH value, were developed and proved stable under an accelerated phase separation test. The textural analysis concluded that the increasing oleogel fraction affected only the spreadability and firmness of blank bigels without significantly changing their cohesiveness and adhesiveness. However, the same conclusion turned out to be inapplicable for the nanodispersion-loaded semisolids, in which an increase in the lipophilic portion altered all parameters studied. The relationship between the dynamic viscosity and the shear rate, determined through rheological studies, ascertained the shear-thinning behavior of all semisolids obtained. The hydrogel-to-oleogel ratio of 80:20 was considered a turning point at which a formulation with optimal consistency and sufficient structural integrity was elaborated. With regard to ease of application and the preferable behavior during topical administration, HP-NLC-BG2 was chosen as a vehicle for NLC-encapsulated HP-rich SJW extract. 

The wound-healing properties of the selected bigel were investigated in vivo on Wistar male rats by treating an incised wound. The tensile strength test was employed to measure the therapeutic effect; the latter was compared with that of a commercial herbal semisolid and negative control. The highest force required to rupture the healed wound was reported for the animals treated with HP-NLC-BG2; proving its superior therapeutic effect.

According to the results of all investigations, NLCs may be considered a promising colloidal system for preserving HP in SJW extract. Regarding topical application, bigels may serve as a suitable vehicle, unfolding the phytochemical’s wound-healing potential. Further investigations concerning HP stability and degradation kinetics would benefit the general scientific public and wound-healing management.

## 4. Materials and Methods

### 4.1. Materials

The materials used in the study, including SJW air-dried ground flowers, leaves, and shoots (Bilec Company, Troyan, Bulgaria), anhydrous methylene chloride (≥99.7%, Thermo Fisher Scientific, Waltham, MA, USA), methanol (≥99.9%, analytical grade, Thermo Fisher Scientific, Waltham, MA, USA), HP standard (≥85%, HPLC grade, Merck KGaA, Darmstadt, Germany), acetonitrile (>99.8%, HPLC grade, Thermo Fisher Scientific, Waltham, MA, USA), phosphoric acid (HPLC grade, Thermo Fisher Scientific, Waltham, MA, USA), double-distilled water (Gesellschaft für Labortechnik GmbH, Burgwedel, Germany; DDW), AO (Alteya Organics, Stara Zagora, Bulgaria), BO (Alteya Organics, Stara Zagora, Bulgaria), PSMO (Sigma-Aldrich, St. Louis, MO, USA), SMO (Thermo Fisher Scientific, Waltham, MA, USA), PM407 (Kolliphor^®^ P407; Sigma-Aldrich, St. Louis, MO, USA), SMS (Thermo Fisher Scientific, Waltham, MA, USA), ketamine 5% (Bremer Pharma GmbH, Warburg, Germany), xylazine 2% (Alfasan Int., Woerden, Netherlands), Jodseptadon 10% (Chemax Pharma Ltd., Sofia, Bulgaria), and sodium chloride 0.9% solution (B. Braun Melsungen AG, Melsungen, Germany), were of pharmaceutical grade. GB (Compritol^®^ ATO 888) was a gift from Gattefossé, Saint-Priest, France. A commercial supercritical CO_2_ extract of SJW with an HP concentration of 40.1% was kindly provided by Flavex Naturextrakte GmbH, Rehlingen-Siersburg, Germany.

### 4.2. Methods

#### 4.2.1. Preparation of the *Hypericum perforatum* L. Extracts

Weighed portions (about 30 g each) of the plant material were introduced into tightly sealable 250 mL borosilicate bottles. Anhydrous dichloromethane (CH_2_Cl_2_) was used as the sole extraction solvent. The maceration process was carried out at solid-to-solvent ratios of 30:75 and 30:100 (*w*/*v;* g/mL). The resulting suspensions were initially purged with argon for 0.5 h and then set aside for at least 2 days in the dark. The thus-obtained yellow-green extracts were decanted and filtered under vacuum through sintered glass filters. The used organic solvent was removed under reduced pressure and continuous argon bubbling. The acquired dark green extracts were stored in a freezer at −20 °C.

#### 4.2.2. HPLC Determination of HP

##### Chromatographic Method

The Thermo Scientific UltiMate 3000 Analytical LC System, equipped with a variable wavelength vibration detector (Dionex UltiMate 3000 VWD) and a diode array detector (Dionex UltiMate 3000 DAD−3000 Diode Array Detector) (Thermo Fisher Scientific, Waltham, MA, USA), was used for the HPLC-UV quantification of HP. The Thermo Scientific HYPERSIL GOLD AQ C18 (150 mm × 4.6 mm, 5 μm) analytical column, protected by an HYPERSIL GOLD AQ C18 (10 mm × 4.6 mm, 5 μm) guard column, was chosen for the separation of the substance in question. Isocratic elution with a mobile phase of 0.3% phosphoric acid and acetonitrile (10:90, *v*/*v*) was performed at a 0.8 mL/min flow rate. The columns and the autosampler temperatures were maintained at 25 °C and 10 °C, respectively. The UV detection was conducted at 273 nm, and the injection volume was 20.0 μL [33].

The relative presence of HP in the obtained extracts was determined using the external standard method. The same method was applied to investigate the commercially available SJW extract. All chromatographic analyses were performed under equal conditions.

##### Standard and Test Solutions Preparation

A standard stock solution of HP in methanol with a concentration of 50.0 μg/mL was prepared and subsequently used to obtain working standard solutions (with concentrations of 50.0, 40.0, 30.0, 20.0, 10.0, and 1.0 μg/mL). A six-point calibration curve was constructed (x = concentration of the standard solutions [μg/mL] and y = peak area [mAU·min]). The proposed by the International Conference on Harmonization (ICH) criteria [60] were used for method validation.

All SJW extracts were dispersed in methanol and diluted to a final concentration of 20.0 μg/mL. Afterward, six replicates of each sample were injected into the HPLC system.

#### 4.2.3. Preparation of NLC Dispersions

Two NLC models containing different liquid lipids (Table 7) were prepared by emulsification, high-shear homogenization, and ultrasonication.

The lipid phase comprising exact amounts of GB, liquid oil, and SMO was heated to 80 ± 2 °C. DDW and PSMO were mixed to form the aqueous phase and heated to the same temperature. The hydrophilic phase was added to the lipid one dropwise under constant stirring at 750 rpm for 3 min. The hot pre-emulsion was further homogenized using an Ultra-Turrax^®^ T25 homogenizer (IKA^®^-Werke, Staufen, Germany) at 10,000 rpm for 3 min. The last preparation step was to sonicate the dispersion for 15 min at ambient temperature (Advantage-Lab™, Fisher Scientific GmbH, Vienna, Austria). 

The HP-rich SJW extract-loaded NLC samples (HP-NLC1 and HP-NLC2, respectively) were obtained under dark conditions by incorporating the extract (1.25%, *w*/*w*) in the lipid phase before the emulsification.

#### 4.2.4. Characterization of the Carriers

##### Particle Size, Polydispersity Index, and Zeta Potential 

The mean hydrodynamic diameter and the PI of NLC and HP-NLC samples were assessed by a photon correlation spectroscopy technique using Zetasizer Ultra (Malvern Panalytical Ltd., Malvern, UK). All nanodispersions were diluted 1000-fold to obtain a suitable measurement density. The analyses were performed at 25 °C in a backscattering mode. The nanoparticles’ ZP was investigated via laser Doppler electrophoresis using the same apparatus. The indicated parameters were determined immediately after nanocarrier preparation and after 1 month of storage at 4 °C. The results after three measurements were expressed as the mean ± SD.

##### Entrapment Efficiency Determination

The EE of the extract-loaded carriers was estimated by a direct method as follows: 1.00 ± 0.01 g of each HP-NLC dispersion was centrifuged using a microcentrifuge (D2012 Plus, DLAB Scientific, Rowland St. City of Industry, CA, USA) at 10,000 rpm for 30 min. The separated pellets were washed three times with DDW, dispersed in methanol, and filtered through a Minisart^®^ RC25 syringe filter with a 0.20 μm pore size (Sartorius, Göttingen, Germany). The evaluated HP amount (m_encapsulated HP_) and the initial one (m_total HP_) were used to calculate the EE according to the following equation:

(1) $$EE(\%)=\frac{m_{encapsulated\;HP}}{m_{total\;HP}}\times 100.$$

##### ATR-FTIR Spectroscopy

The ATR-FTIR spectra were collected at room temperature using a Tensor II FTIR spectrophotometer (Bruker, Bremen, Germany). Each spectrum was taken as an average of 32 scans. The OPUS 8.0 software was used to autocorrect the spectral baselines. The colloidal samples were studied after evaporation of the aqueous medium to obtain dry, thin films. 

##### X-ray Diffraction Analysis

The HP-rich SJW extract, the solid lipid, the lipid blends (solid-to-liquid lipid ratio of 7:3), as well as NLC and HP-NLC samples, were analyzed by XRD in a Panalytical Empyrean diffractometer (Malvern Panalytical Ltd., Malvern, UK). The diffractograms were obtained using a Cu-Kα source (λ = 1.5406 Å). The measurements were carried out at a 1 s/step scanning rate with a step size of 0.013°.

##### Transmission Electron Microscopy Characterization

TEM was utilized to investigate the size and inner morphology of the blank and extract-loaded nanoparticles. Briefly, after gentle shaking, the nanodispersions were deposited on carbon-coated Cu-grids and air-dried for 24 h prior to the analysis. The micrographs were evaluated using HRTEM JEOL JEM 2100 (JEOL Ltd., Tokyo, Japan) with an accelerating voltage of 200 kV. 

#### 4.2.5. Preparation of Bigels

Four blank bigels comprising different hydrogel-to-oleogel ratios were prepared: BG1 (90:10), BG2 (80:20), BG3 (70:30), and BG4 (60:40). The hydrogel was obtained by dissolving PM407 in DDW (in a concentration of 20%, *w*/*w*) for 24 h at 4 °C. The resulting clear polymeric solution was equilibrated to room temperature to form a transparent gel. The organogel was prepared by mixing SMS (15%, *w*/*w*) and BO heated to 60 ± 2 °C under moderate stirring (200 rpm) until the complete dissolution of the gelling agent. The hot organogel was added portion-wise to the hydrogel under mechanical stirring (IKA^®^ EUROSTAR 60 digital, IKA^®^-Werke, Staufen, Germany) at 1000 rpm for 10 min. 

HP-NLC-containing bigels (HP-NLC-BG1, HP-NLC-BG2, HP-NLC-BG3, and HP-NLC-BG4, respectively) were prepared following the same procedure and ratios as the blank semisolids. The hydrogel medium was obtained by mixing a certain amount of the selected HP-NLC dispersion with DDW to provide a 0.5% (*w*/*w*) extract concentration in the final formulations.

#### 4.2.6. Studies on the Semisolids

##### Physical Appearance

The bigels were visually inspected for their organoleptic properties: color, odor, homogeneity, and consistency. The formation of a semisolid structure was tested using the tube inversion method [18].

##### Optical Microscopy

The structural features of the biphasic formulations were analyzed using an optical microscopic technique. An accurately weighed amount of each bigel was diluted in a 0.5% (*w*/*w*) methylene blue solution to a final concentration of 0.5% (*w*/*w*). Then, the samples were subjected to ultrasonication for 5 min to obtain homogeneous emulsions. Next, 10 μL aliquots were dripped on a microscope slide and examined at 40× magnification. An optical microscope (Leica DM1000, Leica Microsystems, Wetzlar, Germany) equipped with a Leica ICC50W camera and the associated Leica Application Suite ver. 3.4.0 software was used for the analysis.

##### pH Determination

The degree of acidity of the semisolids was assessed by measuring the pH value of a 10% dispersion using a portable pH meter (pH 70 Vio, XS Instruments, Carpi, Italy). All measurements were performed three times. 

##### Centrifugation Test 

The stability of the formulated bigels was tested using a microcentrifuge (D2012 Plus, DLAB Scientific, Rowland St. City of Industry, CA, USA) as previously described [61]. About 1 g of each sample was placed in a 2 mL vial and centrifuged at 4000 rpm for 10 min, followed by another cycle at 5000 rpm for the same time. All studies were performed at ambient temperature.

##### Spreadability

The parallel-plate method was used to determine the spreadability of the semisolids [48]. Briefly, 1.0 ± 0.1 g of each formulation was pressed between two glass plates, with the upper one having a mass of 125.0 g, for 1 min. The results were presented as the threefold measured spreading diameter (Ø).

##### Texture Analysis

The mechanical properties of the biphasic formulations (hardness, cohesiveness, and adhesiveness) were investigated by performing a single compression test. A Belle texture analyzer (Agrosta Overseas, Serqueux, France), equipped with a cylindrical probe (18 mm in diameter), was used in the study. The selected pretest and test speeds were 3 mm/s, and the insertion depth was 5 mm. All measurements were performed in triplicate.

##### Rheological Studies

The rheological measurements were performed at 20 ± 1 °C using HAAKE™ Viscotester™ 550 (Thermo Fisher Scientific, Waltham, MA, USA). Triplicate analyses were conducted in an SV DIN coaxial cylinder sensor at shear rates ranging from 0.0123 s^−1^ to 1000 s^−1^. The data for the shear stress as a function of the share rate for each bigel were examined. Three linear and nonlinear models were used to obtain the main rheological parameters, as given in Table 8. The mathematical modeling was completed in the application software of the instrument.

#### 4.2.7. Evaluation of Wound-Healing Potential

##### Experimental Animals

The animal studies were conducted after the approval of the Commission for Ethical Treatment of Animals at the Bulgarian Food Safety Agency (permit number: 265/02.06.2020). All experiments were under the EU Directive 2010/63/EU for animal experiments, the Basel Declaration, and the International Council for Laboratory Animal Science ethical guidelines for researchers [63,64,65]. 

In the experiments, 21 male Wistar rats, each having a body weight of 200–250 g, were used. The animals were situated in the Vivarium of Medical University of Varna and housed in plastic cages under standard environmental conditions: 22 ± 1 °C with a relative humidity of about 55% and 12 h light/dark cycles. They were submitted to a standard pellet diet, with free access to water throughout the experiment.

##### In Vivo Study

The experimental animals were anesthetized by intramuscular injection of 35.0 mg/kg ketamine (5%) and 5.0 mg/kg xylazine (2%), and the skin of the dorsal region of each rat was prepared for aseptic surgery. Two full-thickness paravertebral incisions, each 5 cm long, were made bilaterally at 1.5 cm from the sagittal plane of each animal. They were subsequently sewn with three interrupted surgical sutures and cleaned daily with saline.

Three experimental groups, each containing seven animals, were randomly formed: an untreated, negative control group (A); a positive group treated with a marketed herbal semisolid formulation containing extracts from *Aloe vera*, *Prunus amygdalus*, *Vitex negundo*, and *Rubia cordifolia* (B); a group treated with HP-NLC-BG2 (C). The semisolid formulations were applied topically once a day for a 10 day research period. The sutures were removed on the ninth day; on the next day, the wound-healing effect was determined through the force required to disrupt the repaired skin integrity. The tensile strength test was performed using a tensiometer (Halda Haldex AB 150, Jonard Tools, Elmsford, NY, USA) following the construction of a calibration model to establish the range of values measured (y = 0.2086x, R^2^ = 0.9991). Results were presented as the mean ± SD of a sevenfold analysis [33].

#### 4.2.8. Statistical Analysis

The data on all measurements were processed to obtain the mean value and SD. Analysis of variance was performed to compare the means with a significance level of *p* < 0.05 (SPSS ver. 26.0 software, IBM Corp., Armonk, NY, USA). A subsequent Duncan test for multiple comparisons was used to determine significant differences in the spreadability, firmness, cohesiveness, adhesiveness, and pH values.

## Figures and Tables

**Table 1:** Results of Z-average, polydispersity index (PI), zeta potential (ZP), and entrapment efficiency (EE) tests for the blank and HP-rich SJW extract-loaded NLCs (HP-NLCs). Values are given as the mean ± standard deviation (SD) of three measurements.

Formulation	Days	Z-Average, nm	PI	ZP, mV	EE, %
NLC1	0	125.27 ± 1.36	0.29 ± 0.01	−35.33 ± 1.13	NA
	30	144.93 ± 2.04	0.29 ± 0.01	−33.51 ± 1.07	NA
NLC2	0	133.07 ± 2.30	0.27 ± 0.01	−33.91 ± 1.01	NA
	30	154.94 ± 2.10	0.31 ± 0.03	−33.98 ± 1.19	NA
HP-NLC1	0	142.97 ± 2.00	0.25 ± 0.01	−36.53 ± 3.02	70.44 ± 0.21
	30	212.17 ± 2.76	0.43 ± 0.02	−39.55 ± 3.25	65.61 ± 0.20
HP-NLC2	0	146.00 ± 3.25	0.28 ± 0.02	−36.22 ± 1.68	74.49 ± 0.23
	30	181.13 ± 3.65	0.34 ± 0.03	−42.22 ± 1.36	72.10 ± 0.25

**Table 2:** Degree of acidity of the investigated bigels (mean ± SD, *n* = 3).

	BG1	BG2	BG3	HP-NLC-BG1	HP-NLC-BG2	HP-NLC-BG3
pH value	6.79 ± 0.07 ^a^	6.81 ± 0.05 ^a^	6.82 ± 0.01 ^a^	6.05 ± 0.01 ^b^	6.04 ± 0.01 ^b^	6.05 ± 0.03 ^b^

Means in a column with a common superscript letter (^a,b^) differ (*p* < 0.05) as analyzed by one-way ANOVA.

**Table 3:** Mechanical properties of the biphasic semisolids (mean ± SD, *n* = 3).

Formulation	Spreadability, mm	Firmness, g	Cohesiveness, g·s	Adhesiveness, g·s
BG1	31.50 ± 0.89 ^a^	71.07 ± 0.09 ^e^	86.27 ± 1.25 ^d^	−14.17 ± 0.63 ^a^
BG2	30.17 ± 0.42 ^a,b^	74.67 ± 0.94 ^e^	90.37 ± 1.20 ^d^	−16.03 ± 0.31 ^a^
BG3	29.00 ± 0.41 ^b,c^	81.00 ± 2.94 ^d^	93.7 ± 3.77 ^d^	−15.13 ± 1.03 ^a^
HP-NLC-BG1	28.17 ± 0.12 ^c,d^	112.33 ± 4.71 ^c^	138.8 ± 9.01 ^c^	−19.37 ± 0.90 ^b^
HP-NLC-BG2	26.83 ± 0.96 ^d^	137.33 ± 3.77 ^b^	167.6 ± 8.71 ^b^	−25.10 ± 0.37 ^c^
HP-NLC-BG3	24.75 ± 1.08 ^e^	148.03 ± 0.08 ^a^	182.63 ± 0.70 ^a^	−25.17 ± 1.35 ^c^

Means in a column with a common superscript letter (^a–e^) differ (*p* < 0.05) as analyzed by one-way ANOVA.

**Table 4:** Rheological characteristics of the blank bigels, calculated using different mathematical models (mean ± SD, *n* = 3).

Type of Model	BG1	BG2	BG3
Bingham plastic model (BPM)	η_p_ = 0.57 ± 0.04 Pa·s τ_0_ = 354.89 ± 9.1 Pa R^2^ = 0.84	η_p_ = 0.30 ± 0.09 Pa·s τ_0_ = 440.44 ± 22.92 Pa R^2^ = 0.17	η_p_ = 0.77 ± 0.06 Pa·s τ_0_ = 365.43 ± 12.28 Pa R^2^ = 0.78
Power law model (PLM)	K = 322.63 ± 0.1 Pa·s ^*n*^ *n* = 0.11 ± 0.01 R^2^ = 0.88	K = 394.62 ± 17.45 Pa·s ^*n*^ *n* = 0.08 ± 0.01 R^2^ = 0.72	K = 328.76 ± 6.02 Pa·s ^*n*^ *n* = 0.12 ± 0.01 R^2^ = 0.96
Herschel–Bulkley model (HBM)	τ_0_ = 283.59 ± 4.41 Pa K = 37.12 ± 3.25 Pa·s ^*n*^ *n* = 0.38 ± 0.01 R^2^ = 0.99	τ_0_ = 0 ± 0.01 Pa K = 394.72 ± 48.4 Pa·s ^*n*^ *n* = 0.08 ± 0.01 R^2^ = 0.60	τ_0_ = 236.02 ± 8.64 Pa K = 88.59 ± 7.99 Pa·s ^*n*^ *n* = 0.28 ± 0.01 R^2^ = 0.99

**Table 5:** Rheological characteristics of the HP-NLC-loaded bigels, calculated using different mathematical models (mean ± SD, *n* = 3).

Type of Model	HP-NLC-BG1	HP-NLC-BG2	HP-NLC-BG3
BPM	η_p_ = 2.22 ± 0.01 Pa·s τ_0_ = 627.68 ± 8.32 Pa R^2^ = 0.93	η_p_ = 2.61 ± 0.17 Pa·s τ_0_ = 758.79 ± 9.49 Pa R^2^ = 0.86	η_p_ = 3.05 ± 0.15 Pa·s τ_0_ = 778.84 ± 6.57 Pa R^2^ = 0.92
PLM	K = 627.79 ± 14.58 Pa·s ^*n*^ *n* = 0.11 ± 0.01 R^2^ = 0.88	K = 768.86 ± 8.86 Pa·s ^*n*^ *n* = 0.06 ± 0.01 R^2^ = 0.87	K = 768.89 ± 8.86 Pa·s ^*n*^ *n* = 0.06 ± 0.01 R^2^ = 0.87
HBM	τ_0_ = 578.53 ± 2.53 Pa K = 27.09 ± 1.52 Pa·s ^*n*^ *n* = 0.55 ± 0.01 R^2^ = 0.99	τ_0_ = 682.31 ± 5.41 Pa K = 59.90 ± 4.94 Pa·s ^*n*^ *n* = 0.40 ± 0.02 R^2^ = 0.99	τ_0_ = 762.52 ± 8.15 Pa K = 11.35 ± 3.77 Pa·s ^*n*^ *n* = 0.73 ± 0.07 R^2^ = 0.94

**Table 6:** ANOVA of control (A), reference (B), and exploratory (C) groups.

Source of Variation	SS	df	MS	F	*p*-Value	F crit
Between groups A and B	0.060	1	0.060	0.326	0.581	4.965
Within groups A and B	1.849	10	0.185			
Total	1.909	11				
Between groups B and C	48.256	1	48.256	297.428	9.086 × 10^−9^	4.965
Within groups B and C	1.622	10	0.162			
Total	49.879	11				
Between groups A and C	44.907	1	44.907	1121.930	1.329 × 10^−11^	4.965
Within groups A and C	0.400	10	0.040			
Total	45.308	11				

**Table 7:** Composition of the nanostructured lipid carriers.

Formulation	GB (%*w*/*w*)	Liquid Lipid (%*w*/*w*)		SMO (%*w*/*w*)	PSMO (%*w*/*w*)	DDW (%*w*/*w*)
		AO	BO			
NLC1	7	3	−	2	3	85
NLC2		−	3			

**Table 8:** Mathematical models for rheological properties of bigels.

Type of Model	Mathematical Equation
BPM	$\tau =\tau_{0}+\eta_{p}\cdot \gamma$ [62]
PLM	$\tau =K\cdot \gamma^{n}$ [62]
HBM	$\tau =\tau_{0}+K\cdot \gamma^{n}$ [62]

Note: τ, shear stress; γ, shear rate; $\tau_{0}$, yield stress; *K*, consistency index; *n,* power law index; $\eta_{p}$, plastic viscosity.

## Data Availability

Not applicable.
